# Brain shapes of large-bodied, flightless ratites (Aves: Palaeognathae) emerge through distinct developmental allometries

**DOI:** 10.1098/rsos.240765

**Published:** 2024-09-11

**Authors:** Meghan R. Forcellati, Todd L. Green, Akinobu Watanabe

**Affiliations:** ^1^ Department of Ecology, Evolution, and Environmental Biology, Columbia University, New York, NY 10027, USA; ^2^ Richard Gilder Graduate School, American Museum of Natural History, New York, NY 10024, USA; ^3^ Biomedical and Anatomical Sciences, New York Institute of Technology, College of Osteopathic Medicine at Arkansas State University, Jonesboro, AR 72401, USA; ^4^ Department of Anatomy, New York Institute of Technology, College of Osteopathic Medicine, Old Westbury, NY 11568, USA; ^5^ Division of Paleontology, American Museum of Natural History, New York, NY 10024, USA; ^6^ Life Sciences Department, Natural History Museum, London SW7 5BD, UK

**Keywords:** allometry, micro-CT imaging, endocasts, geometric morphometrics, Palaeognathae, ontogeny

## Abstract

Comparative neuroanatomical studies have long debated the role of development in the evolution of novel and disparate brain morphologies. Historically, these studies have emphasized whether evolutionary shifts along conserved or distinct developmental allometric trends cause changes in brain morphologies. However, the degree to which interspecific differences between variably sized taxa originate through modifying developmental allometry remains largely untested. Taxa with disparate brain shapes and sizes thus allow for investigation into how developmental trends contribute to neuroanatomical diversification. Here, we examine a developmental series of large-bodied ratite birds (approx. 60–140 kg). We use three-dimensional geometric morphometrics on cephalic endocasts of common ostriches, emus and southern cassowaries and compare their developmental trajectories with those of the more modestly sized domestic chicken, previously shown to be in the same allometric grade as ratites. The results suggest that ratites and chickens exhibit disparate endocranial shapes not simply accounted for by their size differences. When shape and age are examined, chickens partly exhibit more accelerated and mature brain shapes than ratites of similar size and age. Taken together, our study indicates that disparate brain shapes between these differently sized taxa have emerged from the evolution of distinct developmental allometries, rather than simply following conserved scaling trends.

## 1. Introduction

Identifying the underlying mechanisms which generate neuroanatomical diversity is an enduring goal in comparative neuroanatomy. Seminal works have analysed total brain volume, brain regional volume and body size measurements of adult vertebrate samples in order to investigate whether evolution follows (i) an integrated or concerted pattern, involving changes in clade-wide scaling relationships (allometry) of brains [1–4] or (ii) a modular or mosaic pattern, where individual brain regions evolve quasi-independently from one another potentially due to loss of genetic, developmental, functional and/or spatial constraints on the overall brain morphology [5,6]. Despite being seemingly dichotomous, evidence has supported a combination of both concerted and mosaic trends occurring simultaneously within the same lineages [7–10].

Greater degree of concerted evolution is characterized by similar wholesale allometric changes to the brain across species [1], which could happen through shared developmentally constrained processes or allometric effects acting across the brain. Studying development and evolution together would elucidate whether conserved or distinct ontogenetic and allometric factors contribute to brain morphology divergence between species [11–17]; however, comparative neuroanatomical studies have typically focused on evolutionary allometric patterns across taxa without examining developmental trends. For instance, in bats, carnivorans and primates, body size accounts for vast majority of brain volume variation, with some notable deviations within each clade [13]. Similar analysis on avian brain volume and body size data demonstrated that these two traits are highly correlated across the avian phylogeny, and decoupling of their scaling relationship has contributed to identifying notable brain size differences among major clades [16]. Yet, without developmental sampling, the cause of interspecific allometric trends remains largely unknown.

Most comparative neuroanatomical studies have taken a volume-based approach to studying trait variation of brains. This has produced rich bodies of work which have yielded powerful insights into how brain scaling relationships may evolve, but implementation of geometric morphometric techniques allows for richer characterization of neuroanatomy. Shape can indicate changes that may be overlooked by measuring relative volumes because structures with the same volume can still have different morphologies, which point to the occurrence of important differences in pattern and process. It is worthwhile to investigate whether patterns in brain volumetric changes across lineages are similar to patterns in brain shape changes. For example, if volumetric and shape data both indicate that larger taxa align with smaller taxa in their allometric trends, then we can infer that the brain morphology of larger taxa represents an extension of conserved developmental trajectories. Alternatively, if shape trajectories between larger and smaller taxa differ fundamentally, then their neuroanatomical variation probably arose through evolution of unique developmental allometric trajectories.

In this study, we further elucidate how developmental and evolutionary allometric trajectories underlie brain evolution by sampling an ontogenetic series of taxa with differences in overall size and brain morphology. Among birds, the large, flightless palaeognaths, referred to as ratites (moas, ostriches, rheas, elephant birds, emus, cassowaries and kiwis), are of particular interest as candidates for examining the influence of size on neuroanatomical evolution [16–19]. Ratites are thought to have evolved from volant ancestors resembling the small-bodied Eocene palaeognath *Lithornis*, and then secondarily lost their flight repeatedly across their evolutionary history [20–22]. Even with the advent of modern micro-computed tomography (μCT) methods, most neuroanatomical descriptions of adult palaeognaths were performed during the early twentieth century without using modern CT scan methods (see [23]). In addition, the literature describing palaeognath cranial ontogeny is even more scarce (but see [17,18,24–27]). Studies have found that ratites differed substantially from members of other early diverging birds such as Galliformes in their relative brain volume proportions and in their brain composition [12,28]. In contrast, a recent comparative volumetric analysis more broadly sampling the avian phylogeny identified large, flightless palaeognaths as sharing a statistically similar allometric relationship between brain volume and body size as some non-avian dinosaurs and other early diverging birds, including galliforms such as the domestic chicken *Gallus gallus* [16]. From the former observation, ontogeny of ratite brain morphology would probably be distinct from that of galliforms, in line with regional volume differences [12,28]. From the latter observation, ratite brain morphology might represent a larger end of a continuum along the same developmental and allometric trajectory shared with other early diverging birds with more modest, typical avian body sizes such as chickens [16].

To investigate these possible scenarios, we utilize a comprehensive ontogenetic series of three ratite species: common ostrich (*Struthio camelus*), emu (*Dromaius novaehollandiae*) and southern cassowary (*Casuarius casuarius*), with the avian model system domestic chicken (*G. gallus*) as comparison. We reconstruct endocasts (internal moulds of the brain cavity) from µCT imaging data and provide a new description of the neuroanatomical changes occurring throughout ontogeny in these birds. We then use a high-density geometric morphometric approach to test whether the patterns identified from brain volume and body size data extend to patterns in brain shape, and how developmental trajectories may relate to differences in endocranial shape between adult birds. We refer to endocranial regions by the name of the corresponding neuroanatomical features that are reflected on the surface (e.g. ‘cerebrum’ for impressions of the cerebrum on the endocranial surface).

## 2. Material and methods

### 2.1. Specimens

Sampling included captive-bred late-stage embryonic to adult specimens of 14 *S*. *camelus*, 14 *D. novaehollandiae* and 18 *C*. *casuarius* from the American Museum of Natural History (AMNH; New York, NY, USA), Museum of Osteology (MOO; Oklahoma City, OK, USA) and T. L. Green Research Collection (TLG; Denver, CO, USA). We combined this new sampling with an ontogenetic series of 14 *G*. *gallus* collected from Charles River Laboratory (North Franklin, CT, USA) and originally published in Watanabe *et al.* [29]. A full list of specimens with specific institution, preservation type, age, sex and µCT scanning parameters can be found in the electronic supplementary material, table S1. All specimens had intact skulls with adequate cranial ossification to reconstruct cephalic endocasts. The age range for each taxon is as follows: *S. camelus*, approximately HH39 (embryo) to at least 20 years old; *D. novaehollandiae*, approximately HH40 (embryo) to 16 years old; *C. casuarius*, approximately HH41 (embryo) to 35 years old; *G. gallus*, 1 day old neonate to greater than 8 weeks old. The stages of embryonic ratite specimens were determined using the criteria previously described in Green and Gignac [24]. Because ratite specimens were collected opportunistically, only an age range was given for some specimens and the mid-point of the range was used as the age for these specimens. Age data were recorded and converted to number of days, and this dataset was used to create additional age data that added the incubation periods of each taxon to the age (21 days for *G. gallus*, 48 days for *C. casuarius*, 50 days for *D. novaehollandiae* and 42 days for *S. camelus*), as well as age data standardized for the respective age of sexual maturity (7 months for *G. gallus,* 5 years for *C. casuarius*, 2 years for *D. novaehollandiae* and *S. camelus*) [30,31]. We refer to the sum of the age and incubation period as ‘total age’, and to the total age standardized with respect to age of sexual maturity as ‘standardized age’. An institutional animal care and use protocol was not required for ratites as all specimens were collected and sampled as cadaveric specimens after death. No ratite individuals were harmed or sacrificed for the purpose of this study. *G. gallus* specimens from previously published research were euthanized through cervical dislocation by Charles River Laboratory, following approved ethical standards. This protocol is described in [29] and was approved by the AMNH Institutional Animal Care and Use Committee in 2014 at the time of specimen collection.

### 2.2. Imaging and endocranial reconstructions

The heads of ratite specimens were µCT-scanned on the following institutional systems: (i) a 2010 GE phoenix v|tome|x s240 high-resolution microfocus CT system (General Electric, Fairfield, CT, USA) located in the Microscopy and Imaging Facility of the AMNH, (ii) a Mediso nanoScan PET/CT (Arlington, VA, USA) located in the Genome Research Center at the Cold Spring Harbor Laboratory (CSHL; Woodbury, NY, USA), (iii) a 2012 Nikon XT H 225 ST μCT system (Nikon Metrology, Brighton, MI, USA) located at the Dentsply Research and Development Office (Dentsply; Tulsa, OK, USA), and (iv) a 2018 Nikon XT H 225 ST μCT system located at the Micro-CT Imaging Consortium for Research and Outreach (MICRO; Fayetteville, AR, USA). Electronic supplementary material, table S1 lists scanner used for each specimen. Larger specimens with multiple scans had separate image stacks which were imported into ImageJ (FIJI) v1.53c [32] and fused using the ‘3D Stitching’ function. Full image stacks of ratite data were imported for the virtual segmentation into Dragonfly v. 2021.1.0.977 (Object Research Systems, Inc., Montreal, Canada) and Amira v. 2019.1 (Thermo Fisher Scientific, Waltham, MA, USA). Endocranial segmentation was conducted following recommendations proposed by Balanoff *et al*. [33]. Reconstructed endocasts were then exported as three-dimensional polygon mesh (PLY) files to be further processed. Endocranial reconstructions of the *G. gallus* are from a previous study [29,34].

**Figure 1.**
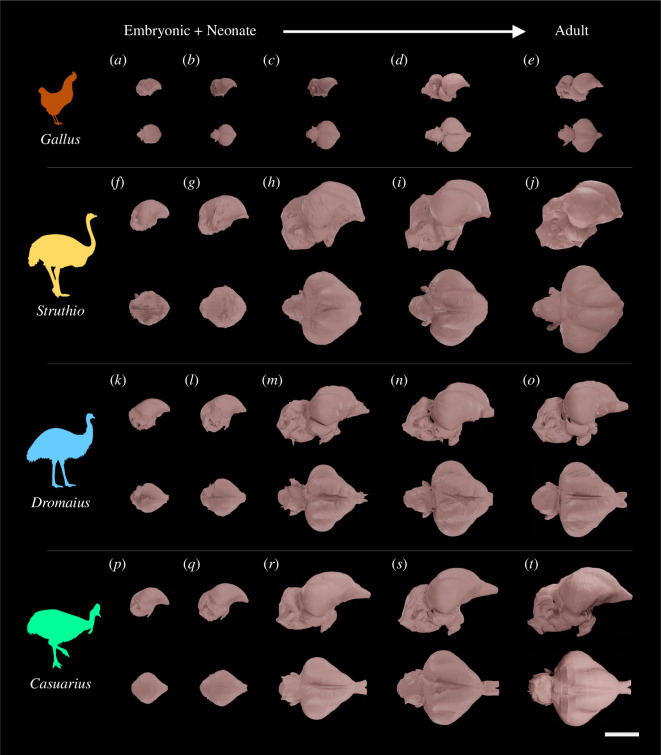
Exemplar endocasts of (*a–e*) *G. gallus*, (*f*–*j*) *S. camelus*, (*k*–*o*) *D. novaehollandiae* and (*p*–*t*) *C. casuarius* sampled for this study in right lateral (above) and dorsal (below) views. Ontogenetic sequences are ordered from most immature (left) to most mature (right) for the following specimens: (*a*) TLG GG015 (immature, 1 day); (*b*) AWRC Gg014 (immature, 1 week); (*c*) AWRC Gg017 (immature, 6 weeks); (*d*) TLG GG017 (immature, 8 weeks); (*e*) AWRC Gg020 (adult); (*f*) TLG SC094 (embryonic, approx. HH39); (*g*) TLG SC032 (immature, 1 day); (*h*) TLG SC030 (immature, 11 months); (*i*) TLG SC083 (adult, approx. 4.0–5.0 years); (*j*) TLG SC080 (adult, approx. 19.5–21.5 years); (*k*) TLG E139 (embryonic, approx. HH40); (*l*) TLG E093 (immature, 5 days); (*m*) TLG E115 (immature, 12 months); (*n*) TLG E114 (adult, greater than or equal to 3.0 years); (*o*) TLG E167 (adult, approx.y 14.8–16.8 years); (*p*) TLG C030 (embryonic, approx. HH41); (q) TLG C024 (immature, 9 days); (*r*) TLGC031 (immature, 14 months); (*s*) TLG C069 (adult, 6.2 years); (*t*) MOO 8031 (adult, 35.7 years). Scale bar = 2 cm.

We considered these to be reasonable proxies of internal moulds of the brain after [29]. Although Watanabe *et al*. [29] did not sample palaeognaths, (i) they found that even in crocodiles, much more distant outgroups to chickens than the ratites we sampled, there was still decent fidelity between the endocranium and the forebrain and midbrain early in ontogeny, (ii) our immature ratites sampled, starting after about 5.5 months in *C. casuarius*, about 11 months in *S. camelus* and about 3 months in *D. novaehollandiae*, preserved impressions of blood vessels on their cerebra throughout the rest of their ontogeny, which other researchers before Watanabe *et al*. [29] used as an indicator of high soft tissue-endocranial correspondence [35], (iii) research in brain volume and skull volume across many species of birds has suggested close correspondence [36], and (iv) we observed that the brain of embryonic *C. casuarius* closely corresponded to the brain cavity (electronic supplementary material, figure S1), in line with the interpretations of Watanabe *et al*. [29]. Thus, we considered brain shape and centroid size (CS) data to closely be reflected by endocranial shape data and we interpret changes in the endocasts as broadly reflecting changes in the brain in the discussion [29,37]. After endocasts were generated, we processed the data in GeoMagic Wrap v. 2020 (3D Systems, Rock Hill, SC, USA). First, we removed small, isolated components from the mesh file using the ‘Mesh Doctor’ tool in the program. Then, we used its ‘QuickSmooth’ tool to globally smooth the endocranial meshes and thus remove noise artificially introduced during segmentation. Research has suggested surface simplification techniques affect a very small fraction of the variation in geometric morphometric shape data for Type I landmarks [38], and it appears this holds true for semi-landmarks as well [39]. After this procedure, to facilitate importation into the software we used for landmarking, we decimated the specimens in Meshlab v. 2022.02 [40] to 1.5 million faces for models with more than this number of polygons (figure 1).

### 2.3. Geometric morphometric data

**Figure 2.**
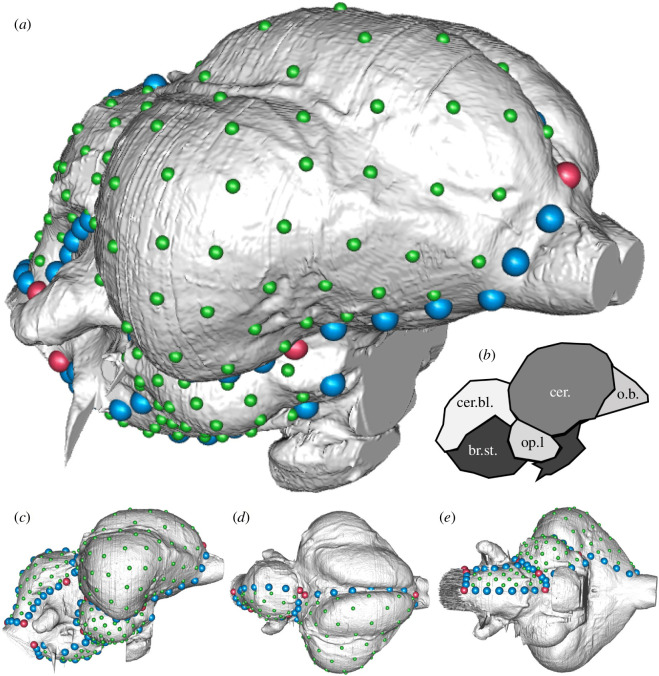
Landmark scheme used in this study shown on the endocranial reconstruction of *D. novaehollandiae* (TLG E054). Red, blue and green points represent fixed, curve and surface landmarks, respectively, in (*a*) right oblique (*c*) lateral, (*d*) dorsal and (*e*) ventral views. (*b*) is a right lateral two-dimensional schematic diagram illustrating the main neuroanatomical region divisions being defined for this study. cer. cerebrum; cer.bl., cerebellum, o.b., olfactory bulb, br.st., brainstem, op.l., optic lobe. Figure is not to scale.

We performed high-density three-dimensional landmark-based geometric morphometrics to characterize endocast shapes, sizes and regional morphologies (figure 2). Our landmarking scheme excludes the olfactory bulb due to the difficulty in consistently identifying and delineating this structure through ontogeny. Instead, we briefly commented on possible differences between taxa in our morphological description of the endocasts. Likewise, we excluded a few important structures from the hindbrain, such as the flocculus and the pituitary gland, because these could not be reliably landmarked. While similar to a previous landmarking scheme [19,29,34], we used a different approach for placing surface semi-landmarks to achieve a more even distribution of surface semi-landmarks than previously. We used Landmark Editor v. 3.6 [41] to place discrete landmarks on the right side of the endocasts and curve semi-landmarks that define the major brain regions. Then, the curve semi-landmarks were resampled using previously customized code [39,42]. For placing surface semi-landmarks, the ‘placePatch’ function from the ‘Morpho’ R package [43] was used to project surface semi-landmarks from a template using a somatically mature domestic *G. gallus*.

After landmark data were collected, we performed generalized Procrustes alignment while minimizing total bending energy and allowing semi-landmarks to slide on the mesh surface using a combination of ‘slider3d’ function in the ‘Morpho’ package [43] and the ‘gpagen’ function in the ‘geomorph’ R package [44–49]. To avoid artefacts from aligning one side of a bilaterally symmetric structure [50,51], we mirrored the right landmarks onto the left sides of the endocasts across the midsagittal landmarks used for our analysis with the ‘mirrorfill’ function from the ‘paleomorph’ R package [52]. After alignment, the mirrored landmarks on the left side of the endocast were removed. The final shape data comprise 13 fixed landmarks, 66 curved sliding semi-landmarks and 140 sliding surface semi-landmarks (electronic electronic supplementary material, table S2). We also acquired CS, the square root of the sum of the squared distances of landmarks from their centroids [53].

### 2.4. Analysis

All analyses were performed using R v. 4.2.1 [54]. We used the ‘gm.prcomp’ function in the ‘geomorph’ R package v. 4.0.6 [48] and base ‘prcomp’ function to perform principal components analysis (PCA) on the endocranial shape data to create morphospaces that visualize overall shape differences across taxa and ontogenetic stages. This was conducted for two datasets: one including all ratite and *G. gallus* specimens, and one with exclusively ratites to target neuroanatomical differences among ratite species.

First, we plotted CS against combined age (postnatal age plus incubation age) to confirm whether ratites exhibit larger brains that go beyond *G. gallus* brain sizes during development. Next, to graphically compare developmental trajectories between taxa, we used regression scores against log-transformed CS. In addition, we used a procedure proposed by Mitteroecker *et al*. [55], that involves a pooled regression of the shape variables on log-transformed CS and age, using the ‘cac’ function from ‘Morpho’ package [43]. For visualizing the differences, we plotted this vector against the first PC of the residual values (RSC1) for each species from that line. This first PC of the residuals captures the deviations from the overall allometric trend shape against log-transformed CS. As in our PCA for studying endocast shape, we repeated this analysis for two subsets of the data: all specimens, and ratites exclusively. We also repeated this analysis using size as the allometric variable and log-transformed total age (postnatal age plus incubation age). Finally, to explore potential mechanisms responsible for variation in developmental patterns, we plotted shape against age standardized by approximate age of sexual maturity for each species, with the acknowledgement that the age of sexual maturity is intraspecifically variable in the taxa we studied.

We next performed non-parametric multivariate analysis of variance (MANOVA) to elucidate group-level differences between nested pairs of taxa [49,56,57]. We did not perform statistical analyses with phylogenetic correction due to relatively poor understanding of the timing of evolutionary divergences among ratite species. Instead, we compared ontogenetic trajectories between nested phylogenetic pairs of taxa (i.e. *G. gallus* and ratites, *S. camelus* and *D. novaehollandiae* + *C. casuarius*; *D. novaehollandiae* and *C. casuarius*). When using MANOVA, we statistically tested for differences in the trajectories across the entire shape space, not just the RSC1 or regression scores we used to visualize shape differences between species.

For our morphological description, we visualized specimens in Blender v. 2.93.3 (Stichting Blender Foundation, Amsterdam, The Netherlands). We examined the major brain regions in three dimensions and noted any differences in the brain regions, the suture lines and the blood vessel impressions we observed between the species studied.

## 3. Results

### 3.1. Neuroanatomical description

#### 3.1.1. Endocranial size

From 1 day old to over 8 weeks old, *G. gallus* endocasts nearly doubled in rostrocaudal length, from approximately 16.7 mm in the youngest specimen (figure 1*a*) to 28.6 mm in the oldest (figure 1*e*). As they progress from embryo to adult, *S. camelus* endocasts also nearly double in rostrocaudal length, from approximately 25.4 mm in the HH39 embryonic specimen (figure 1*f*) to 53.4 mm in the adult over 20 years of age (figure 1*j*). From embryo to adult, *D. novaehollandiae* endocasts nearly double in rostrocaudal length, from approximately 25.3 mm in an HH40 embryo (figure 1*k*) to 55.2 mm in an adult between 14.8 and 16.9 years old (figure 1*o*). From embryo to adulthood, *C. casuarius* endocasts nearly triple in rostrocaudal length, from approximately 23.9 mm in an HH41 embryo specimen (figure 1*p*) to 61.8 mm in the 35.7-year-old adult (figure 1*t*). While the ontogenetic range is not equivalent across taxa sampled in this study, the growth data indicate that cassowaries undergo greater growth during ontogeny.

#### 3.1.2. Forebrain

Across all sampled specimens, the cerebrum expands in each primary anatomical plane during development, and the Wulst becomes more prominent, which previous research has noted undergoes the greatest change in surface area during ontogeny for *S. camelus* [58]. In all taxa, the sulcus between the cerebrum and the cerebellum increases because of increasing distances between the development of the two structures, which starts approximately as the Wulst begins expanding. Adult *S. camelus* differs from *D. novaehollandiae*, *C. casuarius* and *G. gallus* in (i) having proportionately smaller olfactory bulb endocranial regions, a structure that was not characterized in the shape data owing to the difficulty of segmenting it in a systematic and comparable way; (ii) in having slightly more globular cerebra; and (iii) in having greater dorsoventral flexion (which is observed in our shape analysis reported below). *Casuarius casuarius* are unique amongst the sampled taxa in their rostrocaudally elongate and dorsoventrally wide olfactory tract bases (figure 1*t*). This appears to begin at some point between 1.5 and 5.5 months in postnatal development; before this point, *C. casuarius* olfactory bulbs are remarkably similar to those of *D. novaehollandiae* (figure 1*k,l*,*p*,*q*). *Gallus gallus* differ from ratites in having a cerebellum that is more horizontally level with the cerebrum; these two regions are separated by a deep gap which emerges between 6 and 8 weeks of postnatal development, and the difference in positioning of these structures between *G. gallus* and ratites is obvious even from late-embryonic stage (figure 1*a,f*,*k*,*p*).

The species further differ in the development of the Wulst. In adult ratites, the Wulst is more prominent than in adult *G. gallus*. The Wulst also spans a greater relative mediolateral width in *D. novaehollandiae* and *C. casuarius* than in *S. camelus*. Furthermore, it is known that the angle of the left and right Wulsts are distinct between *S. camelus* and the greater rhea (*Rhea americana*) compared with *D. novaehollandiae*, with *S. camelus* and *R. americana* possessing a parallel orientation of the Wulst and *D. novaehollandiae* possessing a caudally divergent configuration [18]. We find that this caudally divergent morphology is also shared between *D. novaehollandiae* and *C. casuarius* (figure 1*m*–o,*r*–*t*). Second, we find that these differences are noticeable starting from when the Wulst first becomes externally visible on the surface of the endocast: in embryonic stages for *S. camelus* and *D. novaehollandiae*, and within the first day post-hatching for *C. casuarius* (figure 1*f,k,p*).

#### 3.1.3. Midbrain

For embryonic and perinatal specimens we examined, it is difficult to distinguish the optic lobe from the cerebrum in lateral view (i.e. figure 1*a,f,k,p*). The optic lobe generally elongates along the rostrocaudal axis in ratites and along the oblique axis in *G. gallus,* where it is ventrally slanted rostrally. The optic lobe is rostrally shifted in ratites compared with that of *G. gallus*. In perinates through to adults, the optic lobe in *G. gallus* is slightly caudal to or at the maximum caudal extent of the cerebrum (figure 1*a–e*). Indeed, its dorsalmost extent wraps around the caudal cerebrum, nearly reaching the sulcus between the cerebrum and cerebellum (figure 1*a–e*). However, in ratites, the optic lobe terminates either at the level of the maximum caudal extent of the cerebrum or rostrally to it (figure 1*f–t*). Another key difference in the optic lobes of *G. gallus* and ratites is the major axis of the ellipsoid contour in lateral view. In the optic lobes of *G. gallus*, it is much more strongly downturned rostroventrally than those of ratites, which are instead angled more horizontally level with respect to the rest of the endocast and the sagittal plane (e.g. figure 1*d*,*i*,*n*,*s*).

#### 3.1.4. Hindbrain

The hindbrain lengthens rostrocaudally during ontogeny for ratites and *G. gallus*. Its relative mediolateral width declines, but this is largely because of the increased lateral prominence of the Wulst and cerebrum and the rostrocaudal lengthening of the brainstem. This lengthening is accompanied by a general loss of convexity of the brainstem, instead becoming more flattened in all specimens. Meanwhile, the flocculus projects farther caudolaterally and becomes more arcuate, as can be seen in dorsal view (e.g. figure 1*a*,*e*).

For all of the endocasts we examined, the cerebellum in the endocast lacks preservation of the cerebellar foliation, despite it being an important characteristic of cerebellar neuroanatomy in birds [59]. This structure failing to preserve in endocasts has been documented in other palaeognaths, and is probably because of a large venous sinus overlying the area which restricts this region from contact with the remainder of the endocast [18,58].

As previously noted, the hindbrain in ratites is ventrally deflected compared with that of *G. gallus*, such that the forebrain is situated relatively more dorsally (figure 1*e*,*j*,*o*,*t*). In *G. gallus*, the dorsal surface of the cerebellum is almost level with the cerebrum (e.g. figure 1*d*). This difference appears to emerge early in development and is already visible between the 1-day-old specimens of *G. gallus* and three perinatal ratites sampled. It becomes more exaggerated in immature chickens as the sulcus between the cerebrum and cerebellum deepens, but slightly less exaggerated in adult *G. gallus* as the Wulst continues expanding dorsally (figure 1*e*). Concomitantly, ratites possess a ventral rotation of the foramen magnum relative to the rest of the endocast: in *G. gallus* this structure angles approximately horizontally with respect to the rest of the endocast, while in ratites, it tends to face more ventrally (figure 1*d*,*i*,*n*,*s*). The shape of the caudal cerebellum varies between taxa in lateral profile. For instance, *G. gallus* specimens possess a shallow but smooth caudal curve of the cerebellum (figure 1*d*,*e*, while it is more linearly slanted in *D. novaehollandiae* (figure 1*m–o*) and vertical and perpendicular in both *S. camelus* and *C. casuarius* (figure 1*h,i*,*r*–*t*).

#### 3.1.5. Sutures

We observed a general pattern of the suture lines for the overlying frontal and parietal bones being poorly visible or not visible at all in embryonic and perinatal endocasts (figure 1*a,f*,*k*,*p*). This is probably because the overlying bones are partially ossified with fontanelles, and because the brain probably occupies proportionately less of the brain cavity in immature individuals [29]. These suture lines then become strongly developed in more mature specimens, with lines clearly delimitating the frontoparietal suture along the boundary between the cerebrum and cerebellum and the caudal parietal suture on the cerebellum visibly prominent in dorsal view of endocasts (figure 1*b,g*,*l*,*q*) [60]. Finally, in adults, these suture lines become much less prominent on the surface of the endocasts probably due to further closure of the suture (figure 1*e,j*,*o*,*t*). Typically, this change tends to occur in the frontoparietal suture first and then the parietal suture. It coincides with both the ossification of these bones and an overall dorsoventral elaboration of brain in the endocast, resulting in the increased development of the Wulst and enlargement of the cerebrum and cerebellum.

### 3.2. Centroid size data

**Figure 3.**
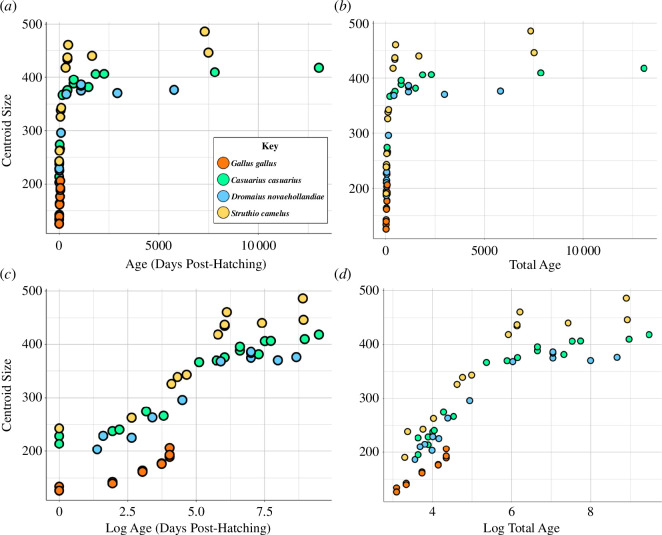
Plots of endocast centroid size against (*a*) days post-hatching, (*c*) log-transformed days post-hatching, (*b*) total (post-hatching age plus incubation age) and (*d*) log-transformed total age between ratites and *G. gallus* throughout ontogeny. Note that ratites (*C. casuarius*, *D. novaehollandiae* and *S. camelus*) encompass endocranial volumes larger than that of *G. gallus* sampled for this study. Common ostriches (*S. camelus*) have the largest adult brains but otherwise generally overlap in size with the other ratites during ontogeny.

Based on CS extracted from our coordinate data, we find that endocasts of ratites surpass the size range observed in the *G. gallus* sampled in this study (figure 3). The youngest specimens of *C. casuarius* and *D. novaehollandiae* overlap slightly with adult *G. gallus* in endocast size. When CS of endocasts are plotted against log-transformed age, the plot indicates that ratites and *G. gallus* have similar slopes but distinct intercepts (figure 3). These results are consistent with the notion that ratites may obtain their larger endocast, and thus, brain sizes, through acceleration or extension of shared developmental scaling trends. Other researchers have similarly found that chickens and ratites are on a common allometric grade in terms of their relative brain-to-body size [12,16,28]. If this is true, we may hypothesize that the youngest ratites should also have similar brain shapes to *G. gallus*, because they may also share a common shape-to-size trajectory. The difference in intercepts could then be explained by longer or accelerated prenatal development, since we only sampled late-embryonic specimens. We evaluate this hypothesis below with shape data.

### 3.3. Shape data

**Figure 4.**
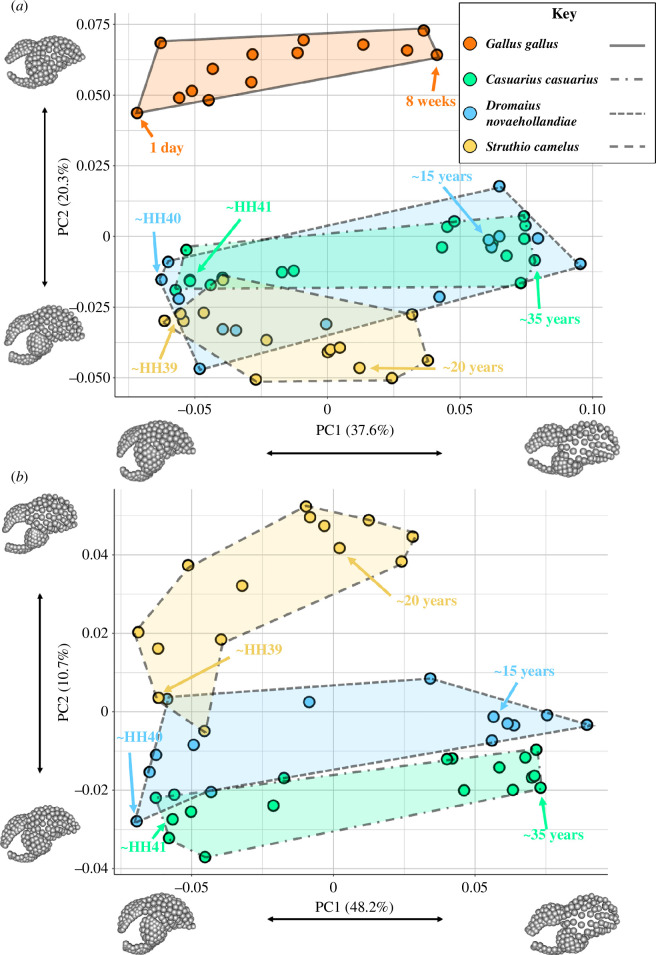
Morphospace of endocast shapes in (*a*) *G. gallus* and ratites; (*b*) within ratites (*C. casuarius*, *D. novaehollandiae* and *S. camelus*). Non-parametric MANOVA shows *G. gallus* and ratites significantly differ in endocast shapes (*n* = 60; *R*^2^ = 0.189; *p* < 0.001). Non-parametric MANOVA shows within ratites, *S. camelus* significantly differs in endocast shape from *D. novaehollandiae* and *C. casuarius* (*n* =46; *R*^2^ = 0.120; *p* < 0.001). In contrast, *D. novaehollandiae* and *C. casuarius* were found with non-parametric MANOVA to lack significant differences in shape (*n* = 32; *R*^2^ = 0.043; *p* = 0.217). Inset images depict extreme shapes along PC1 and PC2 in lateral view. The oldest and youngest specimens of each group, with approximate ages labelled, are indicated with arrows.

We performed PCA on the endocast shape data and created PC morphospaces to visualize patterns of endocast shape variation across the entire ontogenetic series of all taxa (figure 4). For the full dataset, PC1 accounted for 37.6% of the total shape variation, and PC2 accounted for 20.3% of the total variation (figure 4*a*). For ratites exclusively, PC1 and PC2 encompass 48.2% and 10.7% of the total variation, respectively (figure 4*b*). Because the ontogenetic series are oriented along PC1 axes in both morphospaces and a positive correlation between PC1 and log CS (*R*^2^ = 0.493; *p* < 0.0001) exists, we interpreted PC1 as strongly associated with growth, with more mature, larger specimens possessing higher PC1 values and younger specimens possessing lower values along the PC1 axis. This close association between PC1 and size is expected and what has been reported in previous studies examining endocranial shape in birds and non-avian archosaurs [15,17,29,34,61]. The shape changes associated with PC1 include overall rostrocaudal lengthening of the endocast; increased midbrain flexion in the endocast between the cerebrum and cerebellum; ventral shift in the optic lobe and increased separation between it and the cerebrum; rostrocaudal lengthening of the cerebellum and brainstem; and an increased convex curvature of the cerebellum. The PC2 axis separates *G. gallus* and ratites in the full dataset (figure 4*a*), and a more positive PC2 is associated with more rostrocaudally oriented cerebrum and cerebellum; sharper dorsal curvature along the cerebrum and cerebellum; caudal shift in and increased relative size of the optic lobe along with decreased separation between it and the cerebrum; and a more horizontally oriented caudodorsal surface of the cerebellum. The morphospace shows extensive overlap between *S. camelus* and *D. novaehollandiae + C. casuarius* during early ontogenetic stages, but with more divergent morphology along PC2 as they grow (figure 4). PC2 is also associated with log-transformed CS (*R*^2^ = 0.388; *p* < 0.0001), probably driven by the size divergence between *G. gallus* and ratites.

In the ratites-only dataset, changes in PC1 mirror distribution of shape variation identified in the full dataset and similarly are associated with developmental changes occurring in endocast shape (figure 4*b*). *Struthio camelus* occupies a smaller proportion of the PC1 morphospace than the other two taxa. Conversely, PC2 reflects changes associated with differences between the two lineages, with *D. novaehollandiae* and *C. casuarius* sharing similar, although not entirely overlapping, PC2 values, to the exclusion of *S. camelus* throughout all but the earliest stages of their entire ontogeny, during which *S. camelus* overlaps with *D. novaehollandiae* (figure 4*b*). A more positive PC2 is associated with increased prominence of the Wulst, especially rostrally; differences in orientation and position of the optic lobe (both more horizontally oriented with the rest of the forebrain and more ventrally positioned); and a more vertically sloping caudal portion of the hindbrain (figure 4*b*).

Statistically, results from the non-parametric MANOVA on the entire Procrustes shape data (i.e. not PC scores) support the graphical results portrayed by the morphospaces. We statistically tested whether the aggregate shape data across ontogeny for each of these three groups are significantly different from each other. First, *G. gallus* and ratite endocasts differ in shape when their entire ontogenetic sampling is compared (*R*^2^ = 0.189; *p* < 0.001). Similarly, comparing shape variation between *S. camelus* and the combined *D. novaehollandiae* + *C. casuarius* data also resulted in significant differences in the shapes between these two different evolutionary radiations of ratites (*R*^2^ = 0.120; *p* < 0.001). Conversely, our analysis failed to identify significant differences between *D. novaehollandiae* and *C. casuarius* (*R*^2^ = 0.043; *p* = 0.217). As expected by our volumetric data, this suggests that ratites are different from chickens, but also that even within ratites of similar sizes or with convergently evolved similarities in traits (e.g. flightlessness and large body size), there are still neuroanatomical differences, except for emus and southern cassowaries, which are sister taxa.

#### 3.3.1. Developmental trajectories

**Figure 5.**
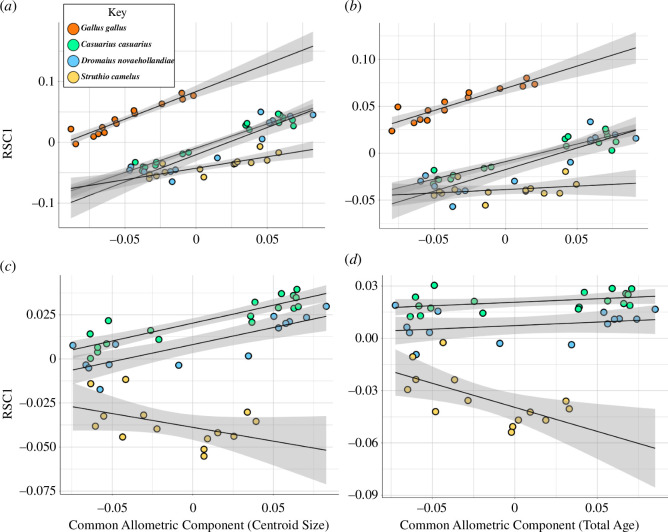
Plots of PC1 of residuals (RSC1) from the common allometric component (CAC) of endocast shape against log-transformed log-centroid size for (*a*) *G. gallus* and ratites and (*c*) only ratites (*C. casuarius*, *D. novaehollandiae*, *S. camelus*), and against log-transformed total age for (*b*) *G. gallus* and ratites and (*d*) only ratites. Grey bands represent 95% confidence intervals for each taxon.

**Figure 6.**
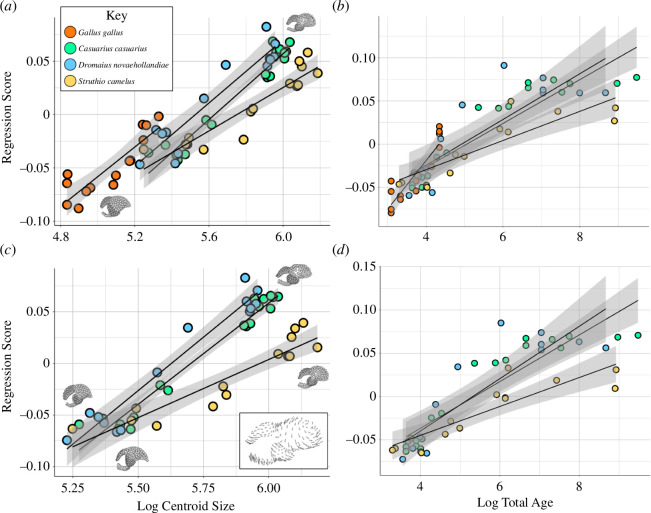
Plots of regression score against log-transformed log-centroid size for (*a*) domestic chickens (*G. gallus*) and ratites (*C. casuarius*, *D. novaehollandiae* and *S. camelus*), with the predicted minimum and maximum shapes along the regression embedded in the figure, (*c*) only ratites, with the predicted minimum and maximum shapes along the regression and the shape changes between *D. novaehollandiae* + *C. casuarius* and *S. camelus* embedded in the figure, log centroid size and against log total age for (*b*) *G. gallus* and ratites, and (*d*) only ratites. The inset figures in (*c*) overlying the regression line represent the predicted minimum and maximum shapes along the pooled regression line of *D*. *novaehollandiae* and *C. casuarius*, while those below reflect the same but for the ostrich regression line. The boxed inset image shows the shape difference between the predicted shape of the largest *C. casuarius* + *D. novaehollandiae* along their pooled regression line (grey spheres) and the predicted shape of largest ostrich along its own regression line (end of black lines).

We graphically compared the ontogenetic trajectories of endocranial shape changes associated with size by plotting the PC1 of residuals (residual shape component, RSC1) from the common allometric component (CAC), or the pooled allometric trends across all the specimens sampled (figure 5). Similar to the morphospace results, we find distinct allometric trajectories between *G. gallus* and ratites (figure 5*a*), as well as between *S. camelus* and the other two ratites (figure 5*c*), but there is some overlap in the ontogenetic shape changes occurring in *C. casuarius* and *D. novaehollandiae* (figure 5*c*). When we visualize the regression score against CS for all the lineages, we find that there is minimal overlap between *G. gallus* and ratites (figure 6*a*,*b*). With this said, we still see considerable differences within ratites, which generally overlap in size with respect to age (figure 6*c*,*d*) but have different shape and size regressions (figures 4–6). Specifically, they all overlap early in development, but *S. camelus* quickly diverges from *D. novaehollandiae* + *C. casuarius*. This can be interpreted as a fundamental difference in the resultant adult brain morphology between the *S. camelus* and *D. novaehollandiae* + *C. casuarius* trajectories, possibly explained by their greater phylogenetic difference. The major differences in trajectories can be visualized from the inset image of figure 6*c*. Here, we can see that *S. camelus* possesses a more globular adult brain morphology, particularly in its cerebrum, and a rostrocaudally compressed cerebellum and optic lobe. When we correct for standardized age (i.e. how old individuals are relative to age of sexual maturity), we see similar results, except that *G. gallus* seems to have a greater slope, indicating accelerated development, probably associated with shorter incubation and life history (electronic supplementary material, figure S2). This result counters the hypothesis that ratites exhibit more ‘mature’ brain morphology compared with *G. gallus* from extending along a shared developmental trajectory.

For our analytical procedure comparing developmental trajectories between *G. gallus* and ratites and within ratites, we find statistically significant differences between taxa at all phylogenetic levels examined (figures 5 and 6). Specifically, we find that ratites are statistically significantly different from *G. gallus* in their developmental trajectories after allometry is accounted for (non-parametric MANOVA: *R*^2^ = 0.175; *p* < 0.001). We similarly find that *S. camelus* is significantly different in its developmental trajectory from *D. novaehollandiae* + *C. casuarius* (non-parametric MANOVA: *R*^2^ = 0.182; *p* < 0.001). Finally, we find that *D. novaehollandiae* and *C. casuarius* are significantly different from each other in their developmental trajectories (non-parametric MANOVA: *R*^2^ = 0.050; *p* < 0.001). When regressing shape over total age (i.e. age including incubation period), non-parametric MANOVA indicates significant correlation between endocast shape and log-transformed age for combined ratites and *G. gallus* (*R*^2^ = 0.273; *p* < 0.001), as well as within ratites (*R*^2^ = 0.343; *p* < 0.001). After the pooled allometric trend is accounted for, *G. gallus* and ratites are shown to exhibit statistically distinct trajectories (*R*^2^ = 0.273; *p* < 0.001), as are *S. camelus* and *D. novaehollandiae* + *C. casuarius* (*R*^2^ = 0.487; *p* = 0.0067).

## 4. Discussion

Our results show that (i) there is a continuous developmental size distribution between brain size in *G. gallus* and ratites, (ii) the brain shapes of ratites are significantly different from each other and *G. gallus* throughout development from late-stage embryos to fully mature individuals, and (iii) the developmental allometry of ratite brain shape is distinct from *G. gallus*, and is not simply an extension of a common shape-to-size scaling relationship. Examining the developmental endocranial shape changes with age reveals that this is partly due to accelerated shape change in *G. gallus* compared with other ratites. As such, based on our endocast data, there is significant dissociation between brain size and age between *G. gallus* and ratites, and even within ratites when comparing *S. camelus* with the clade comprising *C. casuarius + D. novaehollandiae* (figure 3). We also find a significant dissociation between brain shape and age between all the taxa we compared (figures 5 and 6). The pattern of developmental shape changes agrees with the findings of Corfield *et al.* [28,62], which identified differences in brain and body size allometry and total brain to regional brain allometry among adult ratites, suggesting that the shape data are capturing these more regional patterns.

### 4.1. Unique brain shapes in ratites evolved through the acquisition of distinct allometric trajectories

A goal for our study was to identify whether brain shape changes between differently sized taxa—ratites and *G. gallus*—follow an extension of conserved developmental scaling trends, as may be expected by total brain volume and body size relationships [16], or different allometric trajectories, as implied by relative brain volume measurements [12,28,63]. By analysing a densely sampled ontogenetic series of ratites, we find that different endocranial morphologies observed in the adults of these early diverging birds seem to have evolved through the acquisition of distinct developmental allometry. For instance, *S. camelus* differed in its immature and adult endocranial morphology from the other sampled ratites despite largely overlapping in early, postnatal endocast shape and body size (figures 1 and 4). This result implies that the brain morphology of ratites did not evolve simply through increase in size along a shared brain shape-to-size relationship as predicted by concerted brain evolution [1]. In other words, our shape analysis contrasts with the shared allometric trends noted among *G. gallus* and extant ratites based on volumetric data [16]. However, these outcomes do not contradict each other, but are rather complementary because they separately examine evolutionary versus developmental trends and volumetric versus shape trends in allometry. Collectively, the analyses imply that evolutionary allometric trends may be shared across somatically mature *G. gallus* and extant ratites, but the developmental trends leading to evolutionary patterns in shape may be divergent. It is important to note that we specifically obtain this result in the context of shape against endocranial size, rather than incorporating body size into our regression. Studying how these changes map to body size would be valuable for future research.

The relationship between RSC1 and age suggests that ratites and *G. gallus* significantly differ from one another in the way brain shape develops (figure 5). This outcome agrees with the qualitative and quantitative evidence that the brain shape of *G. gallus* and large-bodied ratites are fundamentally different throughout development—the shape of the brain of adult chickens does not converge to that of ratites, even in immature individuals (figure 1). This contradicts the hypothesis that shared brain and body size allometry begets shared brain shape and brain size allometry between these different taxa. In plots of regression scores against log age, the species overlap most closely in the younger part of their shape-size trajectories, such that adult *G. gallus* seem to grade into the ratite regression lines (figure 6). This visual overlap is probably due to an artefact of regression score projection of data. Because there is minimal size overlap and age range overlap between ratites and *G. gallus*, a pooled regression including these two lineages will arrange the shape changes occurring between the smaller, shorter lived *G. gallus* and larger, longer lived ratites along what would appear to be a singular regression line (figure 6). In contrast, it is easy to compare within ratites for this type of a regression given their considerable size and age overlap, and these findings strongly imply divergent morphologies arising through early shifts in developmental shape trajectories.

What may explain the differences observed in brain morphologies between taxa across development? Future research must rule out body size as a confounding factor, but our findings suggest that even species along a common allometric grade which possess similarly large body sizes possess differences in developmental trajectories. For instance, ostriches are thought to have independently evolved their large body size from other ratites, and ratites themselves have large body size variation, even among closely related groups [20,22,64–66]. Naively, by studying allometric grades, one may expect similarly sized, closely related taxa with similarly sized brains to have convergent brain shapes, but our research suggests this is not always the case, as has been suggested by other studies looking at regional brain volume [6,12,13]. Instead, ostriches have distinct brain morphologies and developmental shape trajectories from *C. casuarius + D. novaehollandiae*. This implies that body size alone does not lead to convergent changes in brain shape evolution, even when brain sizes may be similar. Besides body size, the deviation of *S. camelus* from the other two sampled ratites may also be attributable to greater abundance of proliferating neurons found in the telencephalon of adult *S. camelus* brain compared with *D. novaehollandiae* [67].

Another explanation of shape differences observed between taxa may be that *G. gallus*, through domestication, has been selected for accelerated growth and development compared with the ancestral condition, unlike the ratites in our study. Domestication of *G. gallus* is associated with both size-independent and size-dependent brain shape changes relative to red junglefowl, which are considered to resemble the wild-type condition [68]. This means *G. gallus* exhibits derived traits introduced through domestication, although red junglefowl are still fairly similar to *G. gallus* in their brain shape [68]. Furthermore, as a result of their domestication, *G. gallus* have selectively larger cerebra and cerebella than the red jungle fowl when the genetic independence between body size and brain size is taken into account, and these differences apparently emerge through distinct loci rather than through shared genes [69,70]. Sampling red junglefowls in a future study would help corroborate the relationship we have already identified with our dataset and help researchers better understand the relationship between domestication and brain shape and scaling changes.

Interestingly, we find that *C. casuarius* and *D. novaehollandiae* are not significantly different in their brain morphology when comparing across their entire distribution of developmental stages, from late-stage embryo to adult (figure 4), but they are different in their developmental trends (figures 5 and 6). This is a striking finding given that emus and cassowaries are estimated to have diverged approximately 30 million years ago, and possess ecological and behavioural differences [22,30,71–73]. The fossil record of emus and cassowaries is relatively poor, making it difficult to ascertain how their similar endocast shapes relate to body size evolution [22]. For example, if emus and cassowaries evolved large body size independently, then we could investigate the extent to which phylogenetic inertia or their similar allometric trends contributed to their brain evolution, depending on whether the ancestral brain morphology was similar or different in shape. There is greater body size and ecological diversity in extinct casuariids. The oldest *Dromaius* fossil known, *D. arleyekweke*, is estimated to have been less than 20 kg in body size and was probably even more strongly adapted for cursorial lifestyle than modern emus [22,66,74]. Other smaller bodied, (e.g. less than 25 kg), recently extinct emu subspecies (e.g. *D. novaehollandiae minor* and *D. novaehollandiae baudinianus*), extinct cassowaries (*C. lydekkeri*), and extant dwarf cassowaries (*C. bennetti*) also suggest a substantial body size variation within this taxonomic family [22,64–66,74–76]. Because there are few cranial remains from extinct taxa, it may be worthwhile to compare the endocast ontogeny patterns of larger bodied species from the present study with those of smaller bodied *C. bennetti* to study the relationship between brain morphology and body size within this clade [22,64,76,77].

Still, phylogenetic inertia is probably at least a partial reason for brain shape similarities, because the magnitude of differences we observe in endocast shape and developmental trajectories between taxa are concordant with phylogenetic distance, where *Dromaius* and *Casuarius* are sister groups among extant ratites and thus exhibit the closest allometric trajectories and brain morphologies. In contrast to their similar endocast shape, *D. novaehollandiae* and *C. casuarius* possess statistically significantly different developmental trajectories. While statistically significant at the 0.05 level, the results (e.g. relatively low *R*^2^ value) suggest that very similar shape-to-size scaling relationships govern the developmental brain shape changes in *D. novaehollandiae* and *C. casuarius*. Why, besides the aforementioned body size evolution or ecological specialization, are these taxa statistically indistinguishable in gross morphology, but significantly different in their development? The subtle differences between their trajectories, which includes variation in curvature of the dorsal aspect of the cerebrum, could be due to differences in other areas of the skull during ontogeny between these two species, such as the development of an enlarged casque with a neomorphic bony element (median casque element) in *C. casuarius* [24] (figure 6*c*). Species of *Casuarius* are currently the only avian taxa known to exhibit disunited casque development, in which unpaired midline elements participate in casque formation [25]. Notably, certain elements which participate in the *C. casuarius* casque formation, including the paired frontals, form the roof of the forebrain [24,25]. This may explain some of the differences in the thickness of the region immediately caudal to the olfactory bulb (figure 1).

### 4.2. General trends in avian evolution identified by our results and their possible implications for studying non-avian dinosaur evolution

Due to their relatively large and flightless condition, ratites have often been considered analogues to non-avian theropod dinosaurs (e.g. [78]). We observed neuroanatomical traits in developing ratites and *G. gallus* which may yield fruitful hypotheses about how evolutionary changes to the developmental process proceeded along the transition from non-avian theropods to birds. We find that the optic lobes are never caudodorsally positioned with respect to the cerebrum across late-embryonic to adult stages as in non-avialan maniraptorans [79]. What is considered to be the crown bird arrangement of the optic lobes and cerebrum is present even in the youngest embryonic specimens we sampled [79]. This observation corroborates the previous finding that crown birds exhibit distinct allometric trajectories that define their brain evolution and development, compared with non-avian dinosaurs [34]. Conversely, in our late-embryonic and hatchling samples of *S. camelus* and *C. casuarius* (figure 1*a*,*f*,*k*,*p*), the Wulst is barely distinguishable, even as the optic lobe is clearly rostroventrally deflected with respect to the ancestral non-avian condition. This is in line with observations made by prior studies that the Wulst undergoes the greatest proportional amount of size change in development compared with any other part of the endocast in *S. camelus* [58]. This result implies that the Wulst may not be visible in immature bird fossils even if it does in fact emerge in adulthood, possibly obscuring the evolutionary origin of the Wulst when sampling stem birds that are ontogenetically immature, such as certain *Archaeopteryx lithographica* specimens used in other studies (e.g. [80,81]). In other words, we might not expect to be able to identify this structure in stem birds unless they are near or entirely mature specimens.

### 4.3. Future directions

Palaeognaths, while understudied, represent the outgroup to other extant birds (i.e. neognaths), and thus serve to contextualize how avian brain evolution proceeds. In this study, we did not sample all extant palaeognath species, including large-bodied rheas (*Rhea*), the smaller bodied kiwis (*Apteryx*), and the smaller bodied and volant tinamous (*Tinamus*, *Nothocercus*, *Crypturellus*, *Rhynchotus*, *Nothoprocta*, *Nothura*, *Taoniscus*, *Eudromia* and *Tinamotis*). One avenue for further testing whether body size has led to unique brain shapes in the large-bodied ratites would be to include developmental series of kiwis and tinamous in a comparative analysis with our dataset. Because these two palaeognathous groups are both smaller bodied and differ in their volancy, they may provide excellent context for avian neuromorphology and neurodevelopment. However, these taxa are challenging to sample, especially along an ontogenetic series. Also related to our taxonomic sampling, it is worth noting that *G. gallus* is a highly derived lineage that exhibits neurocranial changes introduced as a result of domestication [68]. Therefore, although *G. gallus* is considered an indispensable model organism in the biological sciences and is of modest body size more typical of extant birds, sampling other neognath species along the same allometric grade as the ratites we sampled [16] would clarify whether the pattern observed here extends across the avian phylogeny.

Finally, the birds in our study radically differ in body size and developmental stages, so it is difficult to adjust for this completely in any single regression of their shape. Following up to study how body size directly relates to differences in brain morphology between these taxa would help further elucidate how brain shape developmental allometry may relate to brain volume allometry. For example, developmental series of these taxa are difficult to access, but future studies could sample smaller species of extant cassowaries such as *C. bennetti*, since they exhibit some of the greatest amount of size variation within a genus of palaeognaths [64]. Additionally, although generally endocranial shape and volume tightly correlate with brain shape and volume changes throughout ontogeny in birds, prior research has suggested that for shape this tends to hold out better in the forebrain than in the hindbrain and in later ontogenetic stages in *G. gallus* [29,36]. Likewise, we did not fully quantify all regions of the brain, such as the flocculus, which may be functionally relevant in explaining differences between lifestyles of our taxa (although see [82]). Further sampling these regions would enable us to investigate whether there are other structures in the brain, not quantified by our study, which may be affected by the differences observed between emus and southern cassowaries.

## 5. Conclusions

Our study utilized the large adult size difference between our sampled ratites and *G. gallus* as a case study for investigating whether disparate brain morphologies originate from extension of shared scaling relationships or through different allometric trends. A previous large-scale comparative analysis of avian brain and body sizes suggests that ratites and *G. gallus* fall under the same evolutionary allometric regime [16], but other studies of relative brain volumes have suggested ratites differ substantially from these early diverging birds [12,28,63]. With three-dimensional shape analysis and developmental sampling, we find that the brain morphology, as represented by endocasts, of large-bodied ratites does not conform to the same developmental scaling continuum as *G. gallus*. In other words, these ratites did not acquire their different brain shapes from simply extending the allometric trajectory of *G. gallus* as volumetric data may indicate. Rather, our sampled ratites show a distinct shape–age relationship where shape changes occur more gradually during equivalent age ranges compared with *G. gallus*. This results in distinct allometric trends where *G. gallus* exhibit more mature endocranial shapes for a given size, and through extension of their own distinct shape-to-size trajectory, large-bodied ratites attain their distinct neuroanatomy. Within ratites, *S. camelus* exhibit their own allometric and developmental trajectories that differ significantly from *C. casuarius* and *D. novaehollandiae*. Taken together, our study demonstrates that taxa along the same interspecific allometric continuum do not necessarily overlap in developmental shape allometries. These results oppose our expectation of conserved evolutionary allometry that larger species may follow and extend the developmental trajectory of smaller taxa to obtain different brain morphologies.

## Data Availability

Three-dimensional coordinate data of avian endocasts sampled in this study and code are available via the Dryad Digital Repository [83]. Supplementary material is available online [84].
